# On the different role of alarm substances and fish kairomones in diapause induction in a freshwater planktonic crustacean

**DOI:** 10.1093/plankt/fbac004

**Published:** 2022-02-23

**Authors:** Kazimierz Więski, Mirosław Ślusarczyk

**Affiliations:** West Pomeranian University of Technology, Faculty of Food Sciences and Fisheries, Kazimierza Królewicza 4, 71-550 Szczecin, Poland; Department of Hydrobiology, Faculty of Biology, University of Warsaw at Biological and Chemical Research Centre, Warsaw, Poland

**Keywords:** predation risk, chemical communication, fish kairomones, alarm substances, inducible defenses, diapause

## Abstract

Many aquatic organisms anticipate predation risk via infochemical detection. In a laboratory experiment, we investigated the expression of life-history responses in planktonic *Daphnia magna* under long-lasting exposure to various concentrations of fish kairomones (FK) and alarm substances of *Daphnia* (AS).

*Daphnia* were exposed to one of six concentrations of AS (0, 0.0005, 0.005, 0.05, 0.5, 5 homogenized *D. magna*/L) mixed with the highest concentration of FK, or to one of six concentrations of FK (diluted fish feces of 0, 0.001, 0.002, 0.01, 0.02, 0.1 fish/L) accompanied by the highest concentration of AS. FK alone at the highest concentration were sufficient to induce diapause in 21% of *Daphnia,* while AS alone at the highest concentration did not trigger diapause. Mixed at the highest concentrations, both cues induced diapause in 94% of *Daphnia,* whereas in the control treatment free of infochemicals no ephippial individuals occurred. No significant size or fecundity changes accompanied the diapause response.

The graded type of diapause response observed across a wide concentration AS concentrations suggests that *Daphnia* use AS concentration as a proxy for the level of non-specific predation risk. In contrast, the abrupt change of diapause response across a narrow concentration of FK suggests that they were more critical to identify predator origin than level of risk.

## INTRODUCTION

Chemical cues play a significant role in intraspecific and interspecific communication between organisms in aquatic (Ferrari *et al.*, 2010; Hay, 2009; Scherer and Smee, 2016) and terrestrial (Apfelbach *et al.*, 2005; Parsons *et al.*, 2018) habitats. Among others, they may provide information on other organisms’ proximity, density or behavior, some of which can pose a predatory threat. Two types of infochemicals were recognized for their primary importance in predatory-prey interactions: a) kairomones—water soluble chemicals released unintentionally into the water by a hungry or foraging predator (e.g. skin, urinary or digestive exudates) and b) alarm substances originating from stressed, injured or digested conspecific or heterospecific prey (Ferrari *et al.*, 2010; Mitchell *et al.*, 2017; Parsons *et al.*, 2018). These natural chemicals may control various anti-predatory defense responses: behavioral, morphological, or life-history ones (for a recent review see Scherer and Smee, 2016; Parsons *et al.*, 2018). Kairomones and alarm substances may convey different piece of information on risk of predation. While the response to the alarm cues seems to be innate phenomenon (Chivers and Smith, 1994a, 1994b) significance of predator kairomones may be either innate or learned through association with conspecific alarm cues (Berejikian *et al.*, 1999; Wisenden, 2000). The kairomones may help in identifying a predator given their species specificity. Furthermore their density may signal risk proximity and intensity unless a predator is not interested in a given prey type. In comparison alarm substances may provide a more reliable information about predator’s feeding preferences and activity, but not necessarily about the predator’s identity. Since kairomones and alarm substances may offer complimentary functions, combining information provided by the two cues might allow prey to assess the predation risk with higher precision and should be expected to be widely used by organisms. Surprisingly few defensive reactions were found to be controlled by combined signals at least in aquatic organisms (Scherer and Smee, 2016) including planktonic freshwater crustaceans. Most of the early studies on chemical communication in planktonic organisms have not discriminated between the two cues. Little interest in exploring the combined effect of the two cues might have been caused in part by misinterpretation of the early influential study by Loose and collaborators (Loose *et al*., 1993) showing that diel vertical migration—a predator avoidance behavior—in *Daphnia* may be triggered by fish kairomones alone. The strength of this behavioral response in crustacean prey was observed to be dependent on mere concentration of the predatory kairomones (Loose *et al.*, 1993; Von Elert and Pohnert, 2000). These findings were widely cited, and in consequence, in numerous following studies planktonic organisms were challenged with water derived from predators fed with conspecific prey; therefore it was likely that prey reactions to mixtures of both cues were interpreted as effects of fish kairomones only. More detailed studies indicated that rigorously prepared predator kairomones free of alarm substances may have induced different responses (Pestana *et al.*, 2013; Ślusarczyk, 1999; Stirling, 1995). Over time a consensus has emerged that predatory kairomones may control some reactions (primarily behavioral or morphological defenses) (Loose *et al.*, 1993; Pestana *et al.*, 2013) or alarm cues alone (Pijanowska and Kowalczewski, 1997), triggering a graded response or an on/off switch. Some other prey responses (mostly life–history reactions) might be triggered by a blend of predatory kairomones and prey alarm substances (see Scherer and Smee, 2016 and Mitchell *et al.*, 2017 for the recent review of this issue). This complex situation might be explained by risk/cost constrains. When predation risk is low or announced by unreliable signals (alarm cues or predators kairomones alone), potential prey may employ inexpensive and flexible behavioral defenses, as opposed to when combined signals are present that more reliably inform the origin and intensity of the predation threat, thereby triggering more expensive and inert morphological or life history defenses in the potential prey (Scherer and Smee, 2016).

One of the few defense reactions triggered by complex cues is diapause induction in *Daphnia*, overlooked in recent reviews by Scherer and Smee (2016) and Mitchell *et al.* (2017). The prevalence of this response may be mediated by environmental context that affects overall chances for survival and reproduction of potential prey under risk of fish predation (Ślusarczyk, 2004) e.g. prey body size (Ślusarczyk *et al.*, 2012), food quantity (Ślusarczyk, 2001), temperature (Ślusarczyk and Rybicka, 2011) and refuge sites availability (Ślusarczyk *et al.*, 2005). In *D. magna* production of dormant forms (sexual diapausing eggs covered with protective chitynous envelopes called epphippia) was reported to occur only when fish kairomones and *Daphnia* alarm substances were present simultaneously since each cue separately did not induce the diapause response (Ślusarczyk, 1999). Alarm substances did not have to be digested (modified) by a predator to trigger this reaction with fish kairomones (Ślusarczyk, 1999). The prevalence of the diapause response to infochemicals released by fish fed with *Daphnia* was similar to kairomones of fish fed with other prey species when mixed with homogenized *Daphnia* (Ślusarczyk, 1999). Although the two cues seemed mandatory for diapause induction in *D. magna*, their relative role in this mechanism remained unclear. To address this issue, we explored hereby the predator-induced diapause and some other life-history responses of *D. magna* (size and fecundity changes at first reproduction) in response to prolonged exposition to various concentration mixtures of the two cues.

Few semiochemicals have been identified structurally so far. They are presumed to be volatile or soluble substances of various origin acting in low concentrations alone or as mixtures, making identification particularly challenging (Stirling, 1995). So far, in freshwater organisms, we know the chemical structure of kairomones of herbivorous cladocerans (Uchida *et al.*, 2008; Yasumoto *et al.*, 2005), zooplanktivorous insects (Weiss *et al.*, 2018) and some components of alarm substances of cyprinid fish (Døving, *et al*., 2005; Mathuru *et al*., 2012). A much longer list of infochemicals is waiting for chemical identification, including fish kairomones and alarm cues of crustaceans, which are the scope of the present study. Fish kairomones which induce defense reaction in planktonic cladocerans are believed to be relatively small (<500 Da), non-volatile, polar, and resistant to thermal (−20-120°C), acidic, alkaline or proteinase treatment (Von Elert and Pohnert, 2000) with amine and amide groups (Akkas *et al.*, 2010). Bile acids were recently suggested as the most likely component of fish kairomones (Hahn *et al.*, 2019; Pijanowska *et al.*, 2020). The chemical nature of the alarm substances in crustaceans remains entirely unknown. For the time being, naturally collected infochemicals are typically used in studies on predatory-prey interactions between fish and crustaceans. Water temporary inhabited by fish (Loose *et al.*, 1993) or less often extract of fish feces (Ślusarczyk and Rygielska, 2004) were typically used as the source of fish kairomones while extract of homogenized conspecific crustaceans was utilized as the source of alarm substances (Pijanowska and Kowalczewski, 1997; Ślusarczyk, 1999; Laforsch *et al.*, 2006). Lack of knowledge of their chemical structure has slowed down the pace of research on chemical communication in aquatic systems.

## METHODS

**Fig. 1:**
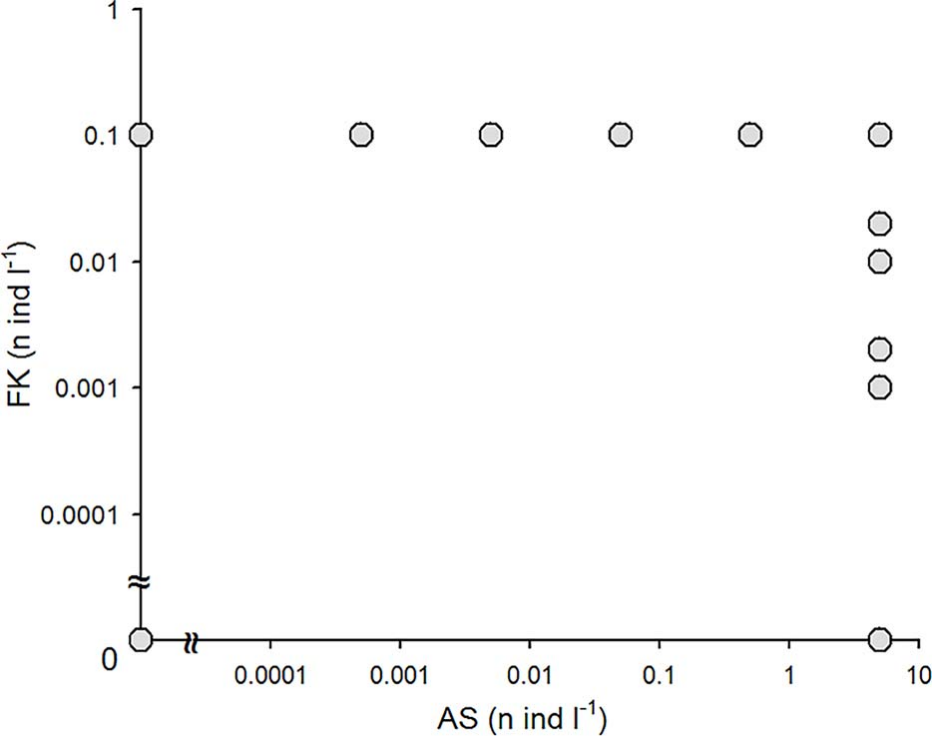
The experimental setup: experimental *Daphnia* were exposed to different concentrations of two infochemicals shown: the FK—fish kairomones made of fish feces, AS—alarm substances made of homogenized daphnia.

The potential role of alarm substances and fish kairomones in the induction of antipredatory defenses were examined in a fractional factorial design laboratory experiment. The experimental setup included two subsets run simultaneously: the first tested effects of six concentrations of alarm substances (0, 0.0005, 0.005, 0.05, 0.5, 5 of homogenized *D. magna* diluted in 1 L of water) accompanied by a high concentration of fish kairomones prepared by dilution of feces of 1 fish in 10 L of water and named after that as a concentration of 0.1 fish per L. The second subset tested six concentrations of fish kairomones (diluted fish feces of—0, 0.001, 0.002, 0.01, 0.02, 0.1 fish per liter of water), with a high concentration of alarm substances (5 homogenized *Daphnia* per liter). The highest concentrations of fish kairomones and alarm cues used in the present study appeared highly effective in the diapause induction in *D. magna* in our former research (Ślusarczyk, 1999). Other researchers typically used comparable or higher concentrations of fish kairomones (Loose *et al.*, 1993; Pestana *et al.*, 2013; Von Elert and Pohnert, 2000) or alarm cues (Pijanowska and Kowalczewski, 1997; Laforsch *et al.*, 2006; Pestana *et al.*, 2013), once more diluted infochemicals appeared ineffective in the induction of various defenses in *Daphnia*. A single control treatment with no cues addition was used as a reference to both subsets (Fig. 1). Each treatment was run in triplicate at 21°C and summer photoperiod (16 L:8D).

### Preparation of chemical cues

As a source of fish kairomones, we used small omnivorous fish *Carrasius carrasius*—that prey on *Daphni*a eagerly. Fish kairomones was prepared from fish feces following slightly modified procedure described by Ślusarczyk and Rygielska (2004). Sixty-nine small crucian carps, *Carassius carassius* (mean wet weight ~ 2 g each) were fed frozen chironomid larvae for 6 weeks to remove any possible former traces of *Daphnia* alarm substances from the fish. On the day the cue was prepared, all fish were fed ca 5 g of chironomid larvae every 45 min, 9 times. Between the feedings, fish feces were collected with a pipette from the bottom of the aquarium and stored in the refrigerator. After 8 hours of collection, the overall feces were homogenized mechanically with a small volume of water, filtered through a 0.45 μm filter and filled with water to form 69 mL of stock solution of fish kairomones. The stock solution was aliquoted in 1 mL doses and frozen immediately (at -20°C) in 1.5 mL Eppendorf vials for further use (each containing extract of 1 fish feces in 1 mL of stock solution). Our earlier experimental study indicated that the extract of fish feces exerts a stronger effect on diapause induction in *Daphnia* than mere water inhabited by fish when applied in relevant concentration (Ślusarczyk and Rygielska, 2004). The extract of fish feces is easier to freeze, store and apply due to much lower volume and higher concentration of fish kairomones compared than fish inhabited water.

The alarm substances were prepared by mechanical homogenization in a small amount of water of ca. 3 000 adult *Daphnia magna* collected from a different location than the experimental clone. The homogenate of *Daphnia* was filtered through a 0.45 μm filter and filled with water to form 60 mL of stock solution of alarm cues. The stock solution was aliquoted in 1 mL doses in 1.5 mL Eppendorf vials (each containing 50 crushed *Daphnia* in 1 mL of stock solution) and kept frozen at –20°C for later use. According to our earlier studies, homogenate of *Daphnia* when combined with kairomones of fish fed with alternative food, exerts a similar effect on diapause induction as relevant number of *Daphnia* digested by fish (Ślusarczyk, 1999).

The two cues were prepared a week before the experiment was started, stored frozen and over the three weeks of the experiment, used daily for the preparation of the experimental media (under the heading Course of the experiment). According to our experience, the two chemical cues retain activity for years when stored frozen (unpublished data).

### Experimental daphnia

The experimental *Daphnia* came from the shallow, euthrophic, fish-inhabited Lake Grosse Binnen, in northern Germany (54^o^33’09”N, 10^o^62’38″E). The experiment was performed on a single clone pre-selected for positive diapause response to a mixture of the fish kairomones and alarm substances. According to our earlier findings, approximately 67% of clones of *D. magna* originated from this lake form diapausing eggs in response to chemical cues on fish predation (Ślusarczyk *et al.*, 2012). Our further studies indicated similar prevalence of diapause response to chemical scents of fish predation in *D. magna* from fish-free and fish-inhabited waterbodies (Ślusarczyk *et al.*, 2013). All experimental animals in the present study were likely genetically identical since they came from a line of sister females reproducing parthenogenetically, derived from a single resting egg that hatched a few weeks before the experiment was started. The mother females of experimental animals (about 60 sister females of similar age) were kept in two 3 L beakers with an aqueous medium exchanged daily and an ambient temperature of 22°C in the presence of a high concentration of algal food (*Tetradesmus obliquus*; > 2 mg C L^−1^).

**Table I:** Summary of the statistics of the factorial models testing interactive effects of two continuous explanatory variables (alarm substances of *Daphnia* - AS and fish kairomones - FK) on the proportion of ephippial females, size at first reproduction—SFR and clutch size of primiparous brood in *D. magna*

Dependent variable	Source of variation	df effec/df residuals	Statistic value	P
Proportion of ephippial females	AS	1/32	Z = 0.41	0.684
Proportion of ephippial females	FK	1/32	Z = 3.72	<0.001
Proportion of ephippial females	AS: FK	1/32	Z = 4.48	<0.001
SFR	AS	1/32	t = −1.50	0.143
SFR	FK	1/32	t = −0.31	0.758
SFR	AS: FK	1/32	t = −0.22	0.824
Clutch size	AS	1/32	t = −1.85	0.074
Clutch size	FK	1/32	t = 0.96	0.345
Clutch size	AS: FK	1/32	t = 0.20	0.843

### Course of the experiment

The control medium was made of lake water filtered through a 0.3 μm filter and supplied with green algae (*Tetradesmus obliquus*) as food for *Daphnia* at a concentration of 0.65 mg C L^−1^ (days 0–4), and 0.7 mg C L^−1^ (after day 4 to satisfy higher demands of larger individuals for food quantity). The lake water was aerated for at least 48 h prior to use to remove infochemicals originating from the lake (Loose *et al.*, 1993). The experimental media were prepared by diluting the defrosted extract of fish kairomones and alarm substances in the control medium. Media were prepared and changed daily. Females of *Daphnia*, randomly chosen from a group of synchronously-hatched offspring derived from the cohort of 60 sister mother females, were placed in experimental beakers within 15 hours of birth. Thirty-two neonates were placed into each 1 L glass jar containing experimental media (30 mL per animal); thus in total, we tested 1 152 experimental females of *Daphnia*. The number of females producing dormant forms (ephippia) were recorded daily in each beaker. Females bearing ephippia were removed without replacement from cultures daily when the media were changed. This procedure allowed for reliable calculations of the proportion of ephippial females in each experimental jar and prevented miscalculation due to a single female’s possible multiple production of dormant forms. Animal densities were maintained constant throughout the reproductive period by reducing the water volume (30 mL per female) when ephippial females were removed. When experimental females deposited their first clutch of subitaneous eggs into the brood chamber, six randomly chosen gravid females from each experimental beaker were measured under a dissecting microscope from the top of the eye to the base of the tail spine (as the measure of size at first reproduction—SFR), and their egg number were determined. Each day newborn offspring were removed from the experimental chambers if they appeared. The experiment ran for 3 weeks, enough time for the production of 5 clutches of offspring per female. As a measure of the diapause response, the cumulative proportion of females producing ephippia was scored in each beaker.

### Statistical analysis

The interactive effects of various concentrations of the two tested infochemicals (considered continuous explanatory variables) on different response variables were tested with factorial analysis of variances and statistical modeling approach. Different types of models and error structures were used for different types of response variables. Data on proportion of ephippial females in experimental beakers were analyzed with generalized linear model (GLM) with applied logit link function and binary error distribution. The data on numbers of females forming and not forming ephippia were transformed prior the analysis with n + 1 transformation to get rid of multiple zero values in most treatments which poses a calculation problem in this type of analysis. The two continuous response variables: the mean body size and fecundity of experimental females at first reproduction in each beaker, were analyzed with multiple regression analysis and two continuous explanatory variables: alarm substances and fish kairomones concentrations.

Moreover, one-way GLM or ANOVA models followed by post hoc pairwise Tukey tests were used to compare the effects of various concentrations of the tested cues (treated as multilevel categorical variables) on the response variables in two experimental subsets separate for each cue. The statistical analysis were performed in R version 3.5.1 statistical program (R Core Team, 2013).

## RESULTS

### Ephippia formation

**Fig. 2:**
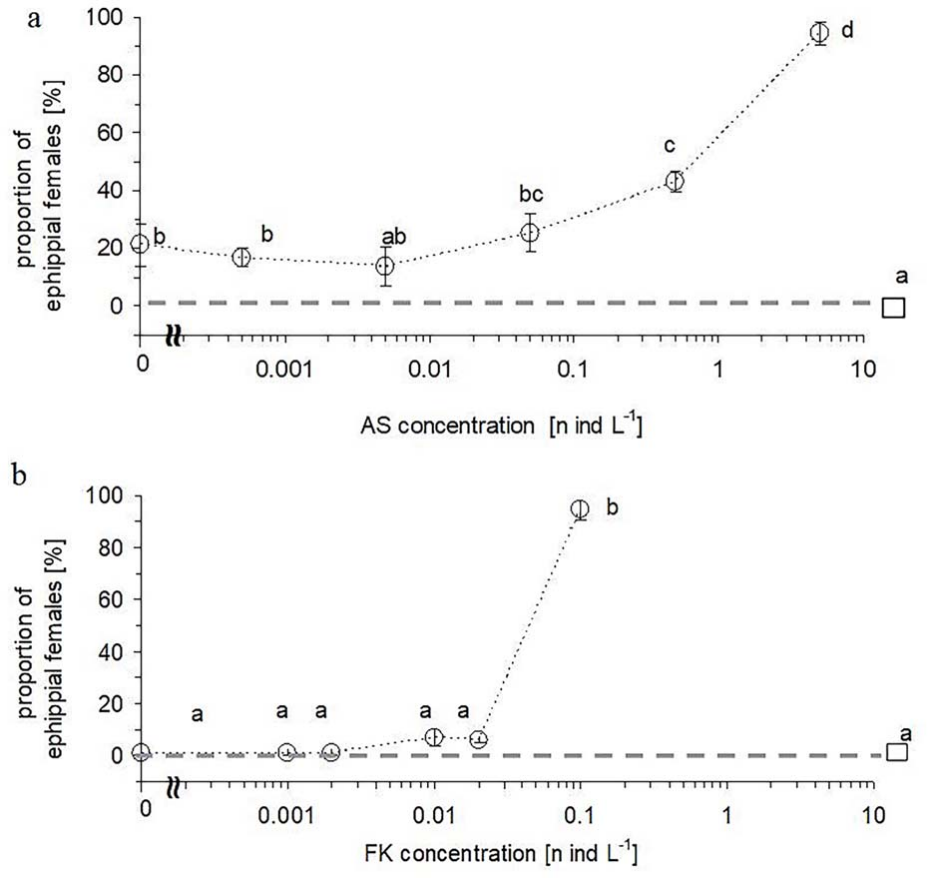
Proportion (mean *±* 1 SE) of *Daphnia* females producing ephippia in the first five broods (circles) when exposed to: a) 6 concentrations of *Daphnia* alarm substances and high concentration of fish kairomones (0.1 fish L^−1^); b) 6 concentrations of fish kairomones and high concentration of *Daphnia* alarm substances (5 daphnia L^−1^). Control treatment with no infochemicals is marked with a square and a horizontal dashed line aside. Letters above symbols indicate homogenous groups of treatments according to post-hoc Tukey test (P ≤ 0.05) measured for two experimental subsets separately.

Fish kairomones and *Daphnia* alarm substances revealed a synergistic effect on ephippia formation by *Daphnia* indicated by significant interaction term between the two continuous explanatory variables (Table I). The presence of fish kairomones alone when at high concentration (0.1 fish L^−1^) was sufficient to induce diapause in 21.1 ± 7.5% (mean ± 1SE) of *Daphnia* females (Fig. 2a). The addition of the alarm substances to the fish medium did not significantly increase the proportion of ephippial females until it reached concentration of 0.5 *Daphnia* L^−1^ that stimulated 43.1 ± 3.6% of females to form ephippia (Fig. 2a). A further increase in concentration of the alarm substances to 5 *Daphnia* L^−1^ resulted in a further increase in the percentage of ephippial females to 94.4 ± 4.0%. As the concentration of alarm substances increased, the first ephippia appeared one brood earlier (data not shown). On the other hand, the presence of *Daphnia* alarm substances alone at high concentration (5 *Daphnia* L^−1^) resulted in a very low proportion of ephippial females (1.1 ± 1.1%) not significantly different from null reported in the control treatment free of both tested cues (Fig. 2b). Addition of fish kairomones to this medium did not stimulate more *Daphnia* females to form ephippia until the highest tested concentration of 0.1 fish L^−1^ was reached that stimulated 94.4 ± 4.0% of females to form ephippia (Fig. 2b). In the presence of both cues, the proportion of ephippial *Daphnia* changed across a wider range of the concentration gradient of *Daphnia* alarm substances than fish kairomones (Fig. 2).

**Fig. 3:**
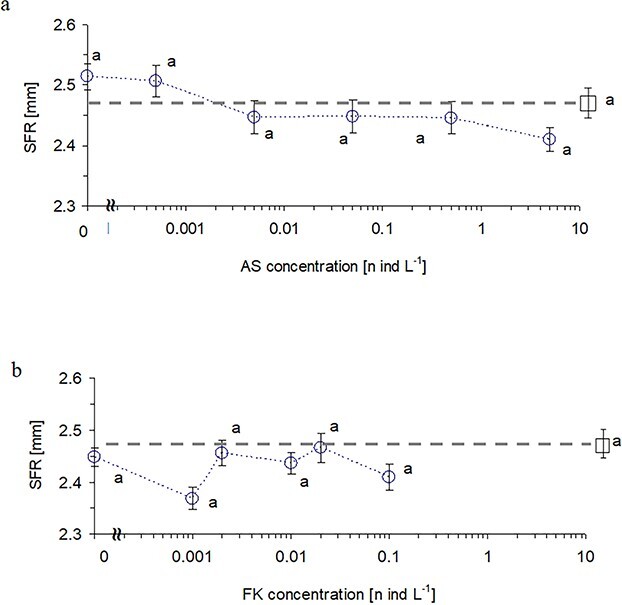
Size at first reproduction (mean ± 1 SE) of *Daphnia* females (circles) when exposed to: a) 6 concentrations of *Daphnia* alarm substances mixed with a high concentration of fish kairomone (0.1 fish/L); b) 6 concentrations of fish kairomones mixed with a high concentration of *Daphnia* alarm substances (5D/L). Control treatment with no infochemicals is marked with a square and a horizontal dashed line aside. Letters above symbols indicate homogenous groups of treatments according to post-hoc Tukey test (P ≤ 0.05) measured for two experimental subsets separately.

### Size at first reproduction

The effect of fish kairomones and alarm substances on size at first reproduction of *Daphnia* was more obscure than on ephippia formation. At the highest concentration of fish kairomones free of alarm substances, the maturation size of experimental females was the highest of all treatments. At a high concentration of fish kairomones with the increase of alarm substances concentration, the SFR of experimental females tended to decrease (Fig. 3a). However, we did not find a significant difference between treatments in pairwise comparisons.

Fish kairomones addition to the alarm cues did not affect maturation size of experimental females either (Fig. 3b). Neither alarm substances nor fish kairomones and their interactions revealed a significant effect on the experimental females’ maturation size (Table I).

### Clutch size

**Fig. 4:**
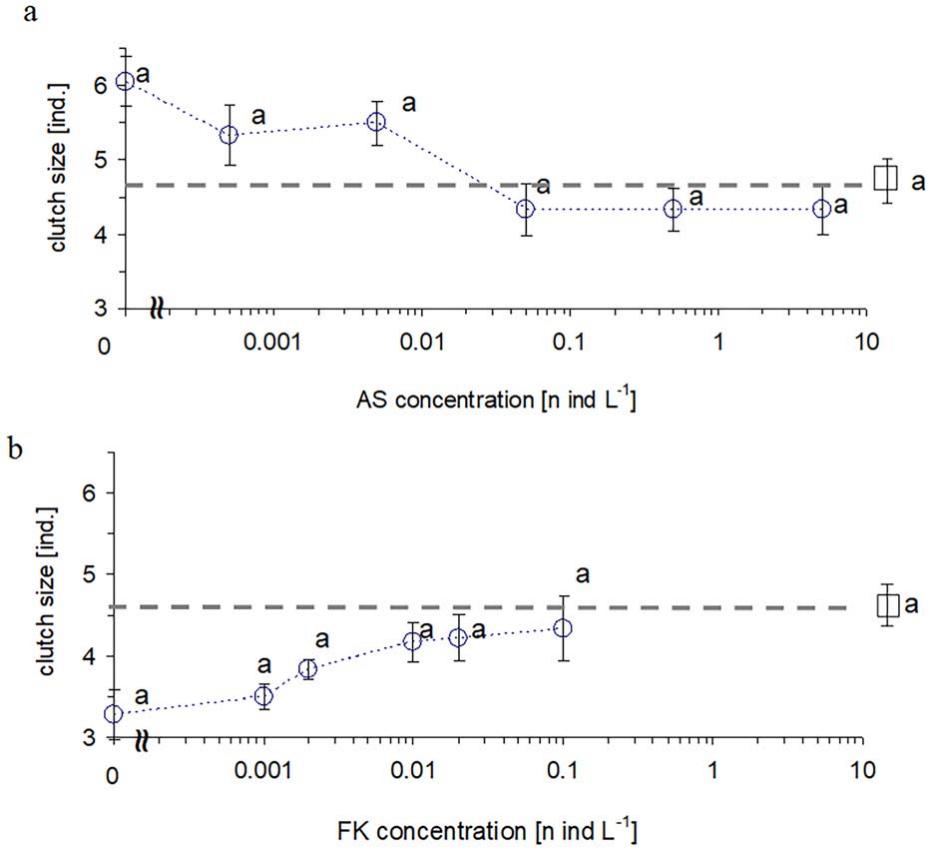
Clutch size (means ±1 SE) of primiparous brood of Daphnia exposed to: a) 6 concentrations of Daphnia alarm substances mixed with a high concentration of fish kairomone (1 fish/10 L); b) 6 concentrations of fish kairomone mixed with a high concentration of *Daphnia* alarm substances (5 D/L). Control treatment with no infochemicals marked with a square and a horizontal dashed line aside. Letters above symbols indicate homogenous groups of treatments according to post-hoc Tukey test (P ≤ 0.05) measured for two experimental subsets separately.

Out of the two tested infochemicals, alarm substances revealed a marginally significant effect on the clutch size of initial broods, unlike fish kairomones or their interactions (Table I). The largest clutch size of the initial brood was observed at a high concentration of fish cues free of alarm substances. An increase in concentration of the alarm substances in this medium tended to cause a stepwise decline in fecundity toward the control level (Fig. 4a). The smallest clutch size was reported at the highest concentration of alarm substances free of fish kairomones. An increase in fish kairomones concentration in that medium tended to gradually increase females’ fecundity toward the control level (Fig. 4b). Multiple pairwise comparisons did not reveal significant differences between treatments (Fig. 4).

## DISCUSSION

Out of three measured life history traits of *Daphnia* commonly associated with anti-predatory defense, the formation of diapausing structures by experimental females appeared most consistent in the present study. While fish kairomones alone when at high concentration (0.1 fish L^−1^) appeared sufficient to induce ephippia formation in a low proportion of *Daphnia* (21.1 ± 7.5%, mean ± 1SE), alarm substances alone even at high concentration (5 crushed *Daphnia* L^−1^) did not cause a significant change in the proportion of ephippial females (1.1 ± 1.1%) compared to the control treatment free of the tested cues with no ephippial females reported (Fig. 2). The two cues, when mixed together, revealed a synergistic effect on the proportion of ephippial females, causing a high prevalence of the diapause response (94.4 ± 0.4%) at the highest tested concentrations of both cues (Table I). Our results may be explained in light of the threat sensitivity hypothesis, which states that in order to diminish the overall defense cost, in a chemical gradient of cues associated with predation, prey can adjust its predator avoidance strategy according to the predation regime (Helfman, 1989). In the case of diapause—the on/off defense response—the magnitude of reaction to the predation risk may manifest in a proportion of the responsive individuals. We observed a more graded type of the diapause response (from 25 to 94% of ephippial females) across a wider concentration of alarm substances (from 0.05 to 5 ind L^−1^), i.e. across two orders of magnitude of their concentrations than in the case of fish kairomones. This might indicate that prey use concentration of the alarm cues to calibrate the magnitude of the predation risk. On the other hand, a relatively abrupt shift from low (6%) to high (94%) proportion of ephippial females that occurred within narrow range concentration of fish kairomones (from 0.02 to 0.1 ind L^−1^) may indicate that kairomones facilitated *Daphnia* in identifying the predator rather than informed it about predation risk (Fig. 2).

There are some differences between the results of the current study and our former study (Ślusarczyk, 1999) concerning the relative effect of kairomones and alarm substances in diapause induction in *Daphnia*. In the former study, the simultaneous presence of both fish kairomones and *Daphnia* alarm substance appeared to be mandatory for inducing diapause in *Daphnia*. In the present study mere presence of fish kairomones as opposed to alarm substance only induced diapause response in low proportion of experimental females (21.1%). The two experiments differed in the sample size of experimental animals and thus “resolution” of our tests: in the current study, we used 96 individuals in total in each treatment, which increased resolution of the frequency response compared to the former study where we only used 10 individuals per treatment. Moreover, chemical signals in both studies were obtained with different methods: the kairomones in the current study originated from fish feces and might have been more concentrated than waterborne cues used in the former study. Interclonal differences in susceptibility to predatory cues may have contributed to the observed discrepancy as well (Boersma *et al.*, 1998; Ślusarczyk *et al.*, 2012) once different clones were used in the two studies. Finally, we can not completely rule out the possibility that the water in the kairomones-only treatment actually contained traces of alarm substances. The lake water was aerated for at least 48 hours to remove natural traces of chemical cues. This procedure was proven to be sufficient to remove fish kairomones (Loose *et al.*, 1993) but might have not been long enough to remove all remains of alarm substances. However, the relatively high concentration of *Daphnia* alarm substances which was required to intensify diapause response in the present study (Fig. 2a) and relatively short half life time of alarm cues reported in some other studies (Stirling, 1995; Wisenden *et al.*, 2009) may indicate this explanation as rather unlikely.

Fish kairomones and alarm substances revealed counteractive effect on *Daphnia* fecundity (Table I, Fig. 4). The highest fecundity was observed in the medium with the highest concentration of fish kairomones free of alarm cues, while the lowest in the medium with the highest concentration of alarm cues free of fish kairomones. The addition of the other cue reduced the original effect for an unclear reason.

The tested infochemicals revealed an ambiguous effect on the maturation size of experimental females. While fish kairomones commonly reduced the maturation size of *Daphnia* in many former studies (for review, see Lass and Spaak, 2003), we did not observe similar effect in our study (Table I, Fig. 3). On the contrary, at the highest concentration of fish kairomones free of alarm substances, the maturation size of experimental females was the highest of all treatments. At a high concentration of fish kairomones with the increase of alarm substances concentration, the SFR of experimental females tended to decrease (Table I). The alarm substances reduced slightly, yet insignificantly, SFR of *Daphnia* (Fig. 3a), confirming former results interpreted as a cautious reaction to an unfamiliar risk (Pijanowska and Kowalczewski, 1997). We can only guess why a strong diapause response to a high level of fish kairomones and alarm substances was not associated with other life history responses (SFR reduction or fecundity enhancement). Our report is not the first one indicating uncoupling of various predatory responses in potential prey. Many former studies reported utilizing one or a few defenses out of an available rage of possible options (Boeing *et al.*, 2006; Boersma *et al.*, 1998; Repka and Pihlajamaa, 1996). While the formation of dormant eggs that are resistant to various environmental extremes, including fish predation (Mellors, 1975), has an apparent adaptive value given high mortality risk of active individuals, a tiny reduction of body size which is commonly observed in *Daphnia* in response to fish kairomones and interpreted as the defense reaction may offer a disputable advantage to potential prey. One may argue that observed minute body size changes do not assure significant protection to potential prey but rather reflect trade-offs in resource investment between growth and reproduction and grater expenses of potential prey on reproduction, thus lower body growth under the threat of fish predation. On the other hand, *Daphnia* are known to produce various inducible morphological appendages that offer more effective protection, such as helmets, neck-teeth, and tail-spines not measured in the present study (Agrawal *et al.*, 1999; Petrusek *et al.*, 2009; Sell, 2000), and trade-off with growth rates (Riessen, 2012; Tollrian, 1995). In addition, behavioral responses to predator presence are likely to cause reduced nutrient uptake and metabolic costs, reflecting growth rates (Dawidowicz and Loose, 1992; Loose and Dawidowicz, 1994).

In the present study, we focused on the effects of different concentrations of fish kairomones and alarm cues in inducting the defense responses in potential prey. We used a single clone of a prey preselected for positive diapause response to those cues to increase the likelihood of the tested reaction. We did not test clonal or species specific variability in the response, which may be the object of further studies. Based on existing data (Boeing *et al.*, 2006; Boersma *et al.*, 1998; Ślusarczyk *et al.*, 2013) we may expect considerable variability in the expression of the defense responses between different couples of prey and predators according to the likehood and strength/consequences of the predatory pressure exerted on the given prey by the given predator. The vast literature on chemical control of predatory defenses details a great variety of effects on life history patterns in prey when exposed to single or multiple cues what makes generalizations particularly difficult (Lass and Spaak, 2003; Mitchell *et al.*, 2017; Parsons *et al.*, 2018; Scherer and Smee, 2016). Our results are not consistent with the experimental data of Reede (1995) who found that some life history responses of *Daphnia galeata* (SFR, AFR) increased gradually with increasing concentration of perch (*Perca fluviatilis*) kairomones when fish were fed with chironomid larvae; however Reede used other species of prey and predator in his study. Our results may be compared with results of Pestana *et al.* (2013) who exposed *D. magna* to different concentrations of alarm cues and kairomones of starved brown trout (*Salmo trutta*). While behavioral and physiological responses of *D. magna* (feeding and respiration) were triggered by single cues (conspecific alarm cues or trout kairomones), life-history shifts were not induced by alarm cues alone. A minor effect of fish kairomones only on life history of *Daphnia* (SFR, AFR, offspring size) was reported. Nevertheless, simultaneous exposure of *D. magna* to fish kairomones and conspecific alarm cues elicited more robust behavioral and life-history responses (Pestana *et al.*, 2013). However, the two studies do no report data on ephippia formation by *Daphnia*.

Behavioral defenses seem less expensive and may be mobilized quickly; they may be controlled by single—less reliable cues. In contrast life history defenses are mobilized or enhanced by the simultaneous presence of both cues (Pestana *et al.*, 2013; present study). As we mentioned in the introduction disregarding unreliable cues may have fatal consequences for organisms; therefore employing inexpensive defenses even at uncertain risk of predation might have a selective advantage. However, more costly life history defenses like diapause or the growth rate (Pestana *et al.*, 2013) may demand more reliable cues for activation. In our study, the mechanism of chemical control of other life-history reactions (SFR, clutch size) must remain unanswered since results appear inconclusive.

In the present study, the diapause in *Daphnia* was triggered by relatively high concentrations of the tested cues, i.e. alarm substances derived from 1 homogenized planktonic *Daphnia* dissolved in 2 to 20 L of water (i.e. 0.5–0.05 *Daphnia*/L) and kairomones derived from feces of 1 fish dissolved in 10 to 50 L of water (i.e. feces of 0.1–0.02 fish/L). These high threshold levels seem more reasonable for *Daphnia* alarm substances than for fish kairomones since *D. magna* mean density reaches 35 individuals/L in the native lake (Lampert, 1991) with unknown fish density. In some reported ponds in the northern hemisphere mean density of *C. carrasius* did not exceed 0.005 individuals/L (Bagge *et al*., 2004) unless in shoals. One possible explanation is that fish kairomones as well as alarm substances degrade at faster rates under laboratory conditions than in nature, as suggested Von Elert and Pohnert (2000). Alternatively, *Daphnia* may experience a high concentration of both cues near the bottom where they commonly hide from fish in shallow waters and where fish feces (i.e. potential source of the cues on predation) may accumulate. The second explanation brings a further question, whether concentration of chemical cues might still be a reliable indicator of predation intensity when concentration of scent of predation and predation risk would not correlate in space and time. All these will cause interpretation problems until we identify the chemical composition of fish kairomones and *Daphnia* alarm substances and learn to measure their concentrations in natural systems.

## CONCLUSION

The synergistic effect of fish feces (presumed source of predatory kairomones) and homogenate of *Daphnia* (presumed source of alarm signals) in the induction of diapause response in experimental *D. magna,* supports hypothesis that both tested cues play a significant role in predatory risk assessment by potential prey. The graded type of diapause response across wide concentration of homogenate of *Daphnia* suggests that the tested animals used conspecific alarm cues to anticipate the magnitude of predation risk. In contrast, abrupt change of diapause response across a narrow concentration of fish kairomones imply that fish kairomones were not used to assess the intensity of the predation risk but rather to identify the predator origin. Surprisingly, the diapause response was not accompanied by other life-history reactions like body size nor fecundity changes, frequently reported in some other studies.

## Supplementary Material

S1_Data_fbac004
