# Supplementary material for: On the different role of alarm substances and fish kairomones in diapause induction in a freshwater planktonic crustacean
Source: J Plankton Res. 2022 Feb 23;44(2):278–87. doi: 10.1093/plankt/fbac004 (PMC9718797; doi:10.1093/plankt/fbac004)
Supplement: S1_Data_fbac004 [file s1_data_fbac004.pdf]

Experimental data used in statistical analysis

treatment	relative Fish kairomones concentration	relative Daphnia alarm substance concentration	mean Daphnia fecundity in each replication (beaker)	mean SFR (mm) of Daphnia in each replication (beaker)	Females with ephippia in each replication (beaker)	Females without ephippia in each replication (beaker)	Proportion of ephippial females in each replication (beaker)
0/0	0	0	5.333	2.526	1	28	0.034
0/0	0	0	5.167	2.523	1	29	0.033
0/0	0	0	3.500	2.404	1	31	0.031
0.1/0	0.1	0	5.000	2.506	8	24	0.250
0.1/0	0.1	0	6.667	2.534	3	26	0.103
0.1/0	0.1	0	6.500	2.505	11	21	0.344
0.1/0.0005	0.1	0.0005	5.000	2.438	4	26	0.133
0.1/0.0005	0.1	0.0005	6.500	2.550	7	23	0.233
0.1/0.0005	0.1	0.0005	4.500	2.533	6	24	0.200
0.1/0.005	0.1	0.005	5.667	2.423	3	27	0.100
0.1/0.005	0.1	0.005	5.167	2.486	3	28	0.097
0.1/0.005	0.1	0.005	5.667	2.431	8	20	0.286
0.1/0.05	0.1	0.05	3.833	2.405	12	20	0.375
0.1/0.05	0.1	0.05	4.167	2.456	5	27	0.156
0.1/0.05	0.1	0.05	5.000	2.484	8	21	0.276
0.1/0.5	0.1	0.5	5.333	2.492	16	16	0.500
0.1/0.5	0.1	0.5	3.500	2.407	12	19	0.387
0.1/0.5	0.1	0.5	4.167	2.439	13	18	0.419
0.1/5	0.1	5	5.000	2.391	31	1	0.969
0.1/5	0.1	5	3.667	2.417	29	2	0.935
0.1/5	0.1	5	4.333	2.422	27	5	0.844
0/5	0	5	3.333	2.485	1	30	0.032
0/5	0	5	3.167	2.472	2	29	0.065
0/5	0	5	3.333	2.388	1	31	0.031
0.001/5	0.001	5	3.333	2.418	1	29	0.033
0.001/5	0.001	5	3.500	2.360	2	27	0.069
0.001/5	0.001	5	3.667	2.329	1	30	0.032
0.002/5	0.002	5	4.167	2.479	1	28	0.034
0.002/5	0.002	5	3.167	2.412	1	31	0.031
0.002/5	0.002	5	4.167	2.478	2	29	0.065
0.01/5	0.01	5	4.167	2.388	2	26	0.071
0.01/5	0.01	5	3.833	2.504	2	29	0.065
0.01/5	0.01	5	4.500	2.418	5	26	0.161
0.02/5	0.02	5	3.333	2.462	3	27	0.100
0.02/5	0.02	5	4.667	2.515	3	26	0.103
0.02/5	0.02	5	4.667	2.421	2	27	0.069
